# High-dose aztreonam potentiates the combined antimicrobial activity of ceftazidime-avibactam against extensively drug-resistant New Delhi metallo-β-lactamase positive *Pseudomonas aeruginosa* in severe pneumonia treatment: the first global case report

**DOI:** 10.3389/fcimb.2026.1786251

**Published:** 2026-03-04

**Authors:** Yifeng Liu, Cuiju Mo, Meng Li

**Affiliations:** 1 Department of Clinical Laboratory, The First Affiliated Hospital of Guangxi Medical University, Nanning, China; 2 Key Laboratory of Clinical Laboratory Medicine of Guangxi Medical University, Education Department of Guangxi Zhuang Autonomous Region, Nanning, China

**Keywords:** aztreonam, ceftazidime-avibactam, combined antimicrobial susceptibility testing, metallo-β-lactamase, *Pseudomonas aeruginosa*, severe pneumonia

## Abstract

Extensively drug-resistant *Pseudomonas aeruginosa* (XDR-PA) present increasing incidence and limited therapeutic options, emerging as one of the serious public health threats worldwide. Here, we report a case of an elderly male patient with multiple primary diseases who developed a severe pneumonia following long-term bedrest due to an accidental tumble after cerebral infarction. In the meantime, plenty of Gram-negative bacilli were repeatedly detected in his sputum and rapidly spread into his blood. This strain was ultimately identified as New Delhi metallo-β-lactamase (NDM) positive XDR-PA. Subsequently, the patient was treated with a combination of ceftazidime-avibactam (2.5 g q8h) and high-dose aztreonam (2 g q6h) under close supervision on his hepatorenal function. No dose adjustment was made throughout the treatment course. After one week of therapy, the patient’s symptoms were remarkably resolved, and finally discharged. This is the first evidence worldwide of successful treatment for severe pneumonia caused by NDM positive XDR-PA using this dose-dependent combination therapeutic regimen as far as we know. The accurate and timely results of carbapenemase typing and combined antimicrobial susceptibility testing support our bold attempt at this novel treatment regimen, ultimately making the patient recover with a favorable prognosis.

## 1 Introduction

Extensively drug-resistant *Pseudomonas aeruginosa* (XDR-PA) has evolved into a major threat to global healthcare systems over the past decades, particularly for patients with advanced age, immune deficiency, and serious conditions. From a global epidemiological perspective, XDR-PA accounts for 15%-30% of clinical *Pseudomonas aeruginosa* (*P. aeruginosa*) isolates from intensive care units, which is mainly driven by the overuse of broad-spectrum antibiotics and cross-transmission (Mirzaei et al., 2020; Montero et al., 2020). In China, the detection rate of XDR-PA has risen from less than 4% to approximately 9% over the past decade (Wang et al., 2021; Yang et al., 2025). More importantly, recent studies reported that the XDR-PA with hypervirulent clone has been sporadically identified around the world (de Paula-Petroli et al., 2018; Zhang et al., 2022). These isolates carry multiple drug resistance genes (e.g., *bla*OXA-902, *bla*OXA-486, and *bla*NDM-1), secrete various virulence factors (e.g., *exoU*, *exoS*, and elastases), and possess strong biofilm formation capabilities, making them difficult to eradicate and contributing to elevated mortality rates, particularly in elderly patients with underlying diseases (Li et al., 2024; Kang et al., 2026).

In general, the combination of ceftazidime-avibactam (CZA) and aztreonam (ATM) medication is recommended as a first-line option against metallo-β-lactamase (MBL) producing *Enterobacteriaceae* by current guidelines (Mantzarlis et al., 2025). However, for XDR-PA, things might not happen so straightforwardly. Besides carbapenemase production, other drug-resistant mechanisms, such as the loss of porin and the overexpression of drug efflux pumps, might be commonly included (Meletis and Karastergiou, 2025). These multifaceted mechanisms not only pose a challenge in therapeutic options but underscore the necessity of antibiotic stewardship and infection control measures to limit the spread of multidrug-resistant strains. On this occasion, the evaluation of the efficacy and optimal dosing regimens against XDR-PA in real−world clinical settings becomes particularly crucial and urgent.

In this study, we report the first case worldwide of successfully treating severe pneumonia caused by New Delhi metallo-β-lactamase (NDM) positive XDR-PA with a combination of CZA and high-dose ATM. This updated therapeutic regimen balances efficacy and safety, offering an individualized and cost-effective option for refractory pulmonary infections among elderly and critically ill populations in future clinical practice.

## 2 Case report

### 2.1 Clinical course

An 83-year-old male patient with a decades-long history of hypertension and chronic obstructive pulmonary disease was admitted to the Emergency Department via stretcher owing to a persistent cough with sputum, accompanied by fever, for over seven months. Four months ago, he accidentally fell down due to right limb weakness caused by the sequelae of cerebral infarction, resulting in multiple rib fractures and pulmonary contusion. Despite previous treatments for pneumonia attributed to prolonged recumbency in multiple local hospitals, his symptoms showed poor clinical improvement. Given the complex and critical nature of his condition, the patient was promptly transferred to the Surgical Intensive Care Unit (SICU) for further management.

Upon admission, the patient remained in a state of moderate coma under sedation. Physical examination was notable for coarse breath sounds with widespread moist rales in both lungs and multiple ecchymoses throughout the body. Non-contrast chest computed tomography (CT) revealed the atelectasis and pleural effusion in bilateral lower lung lobes. Cord-like, patchy, ground-glass, and high-density opacities with ill-defined margins were observed in each lobe of both lungs (**Supplementary Video 1**). The hematological results showed a marked increase in white blood cell count (29.31×10^9^/L) and neutrophil percentage (28.05%), along with reductions in hemoglobin (79.00 g/L) and platelet counts (70.00×10^9^/L), suggesting a severe bacterial infection with the risk of progression to septic shock. Besides, arterial blood gas analysis revealed persistent poor pulmonary oxygenation, with decreased arterial-alveolar oxygen fraction (46.40%) and oxygenation index (290.00 mmHg), despite oxygen administration at 5 L/min.

Therefore, this patient suffered from a chronic pulmonary infection. The accumulated burden of advanced age, multiple primary diseases, and trauma ultimately led to acute exacerbation of his condition.

### 2.2 Microbiological findings

To identify the pathogen responsible for the patient’s severe pneumonia, a sputum sample was collected for bacterial culture on the first day of hospitalization. Abundant Gram-negative bacilli with a slender and straight shape, together with white blood cells, were scattered throughout the sputum smears (**Supplementary Figure 1A**). The strain was ultimately identified as *P. aeruginosa* by matrix-assisted laser desorption ionization-time of flight mass spectrometry (**Supplementary Figures 1B, C**).

According to the results of antimicrobial susceptibility testing (AST), this clinical strain of *P. aeruginosa* displayed an extensively drug-resistant phenotype. With the exception of colistin, all other antimicrobial agents showed resistance. (**Supplementary Table 1**). Notably, the patient rapidly developed a hematogenous dissemination caused by the XDR-PA. During the subsequent stay in the SICU, multiple attempts to eradicate this pathogen were ultimately unsuccessful. On day 4 post-admission, the DNA sequences of *P. aeruginosa* were detected both in the patient’s blood and bronchoalveolar lavage fluid (BALF) by metagenomic next-generation sequencing, with 27,388 and 25,657 reads, respectively.

To elucidate the antibiotic-resistant mechanism of this XDR-PA strain, we first performed a carbapenemase typing assay. As shown in **Supplementary Figures 2A**, the inhibition zone diameter of imipenem increased significantly following the addition of ethylenediaminetetraacetic acid but was unaffected by 3-aminophenylboronic acid. The result of the lateral flow immunochromatographic assay further confirmed the presence of NDM produced by the XDR-PA strain (**Supplementary Figure 2B**).

**Figure 1:**
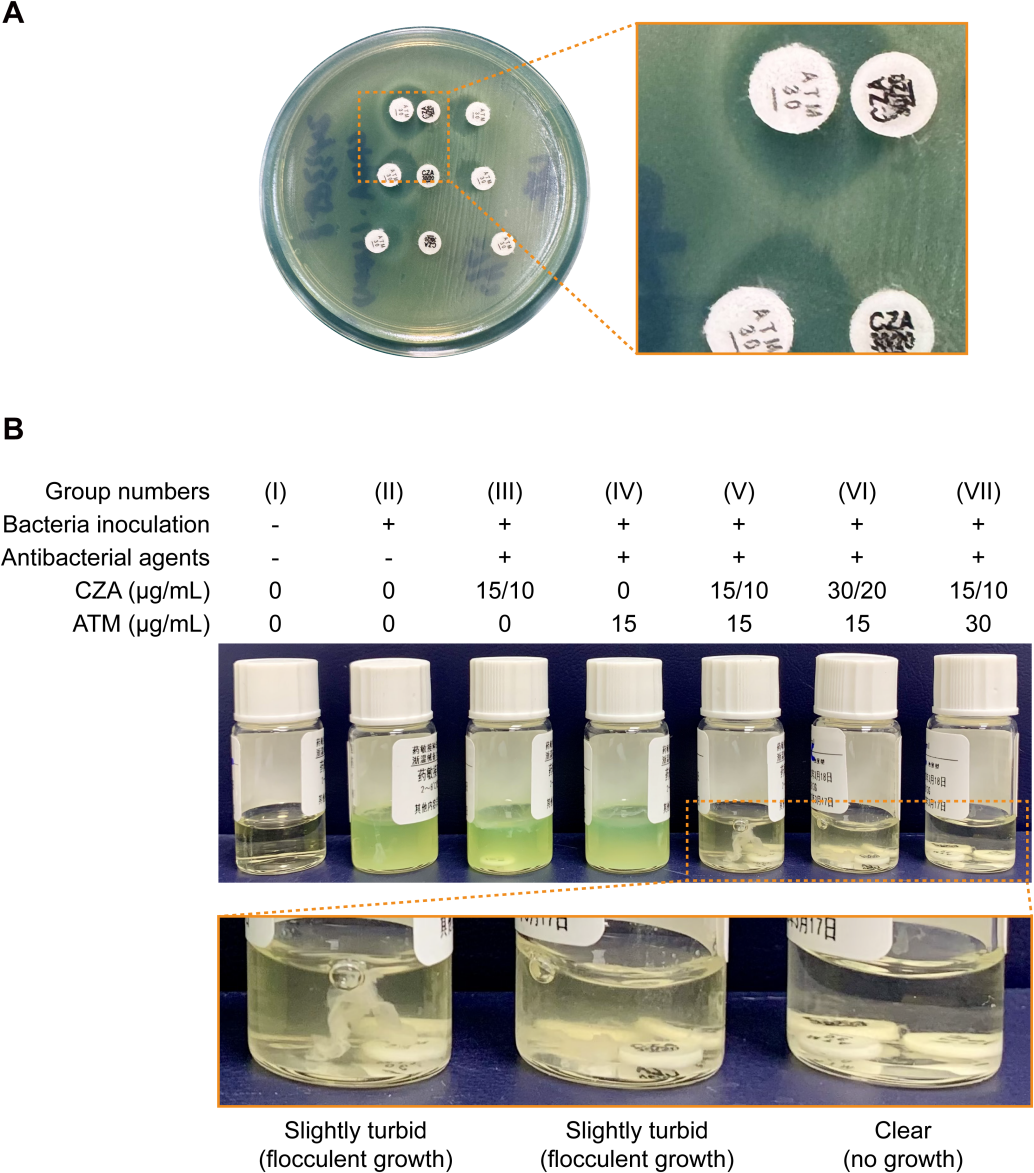
Effects of ceftazidime-avibactam plus aztreonam cocktail on NDM-producing *Pseudomonas aeruginosa in vitro*. **(A)** The disk diffusion test was performed on the Mueller-Hinton agar using ceftazidime-avibactam and aztreonam disks with varied spacing. **(B)** The broth disk elution test was performed in parallel using 2 mL of cation-adjusted Mueller-Hinton broth supplemented with the indicated components and drug concentrations. The partial magnifications show the section marked with a dashed box in detail. Abbreviations: CZA, ceftazidime-avibactam; ATM, aztreonam.

To verify whether the CZA + ATM cocktail exhibits similar efficacy against the NDM positive XDR-PA strain collected from our case as it does against MBL producing *Enterobacterales*, we first conducted the combined AST of CZA and ATM using the double disk diffusion method. As shown in **Figure 1A**, an inhibition zone appeared on the ATM disks on the side adjacent to the CZA disks. The diameter of the inhibition zone increased progressively as the distance between the two disks decreased, indicating a synergistic effect between ATM and CZA. More interestingly, the enlarged diameter of the inhibition zone was also observed between the adjacent ATM disks when the image was zoomed in.

To elucidate the underlying reasons responsible for this phenomenon, we next eluted the antimicrobial agents into cation-adjusted Mueller-Hinton broth at the specified concentrations, followed by inoculation with the XDR-PA strain at a density of 7.5×10^5^ CFU/mL. The resultant mixtures were then incubated at 35 °C for 18 h. As expected, the individual use of CZA or ATM had a limited antimicrobial effect on the NDM positive XDR-PA (**Figure 1B** group III and IV). After the combination of these two drugs, both the bacterial growth and the pyoverdine production were inhibited significantly (**Figure 1B** group V-VII). However, upon closer observation, it became apparent that among all combination therapy groups, only the culture medium containing CZA plus high-dose ATM was distinctly clear (**Figure 1B** group VII). These above results suggest that increasing the ATM dosage enhances the bactericidal effect of CZA + ATM combination on the NDM positive XDR-PA, supporting the leading role of ATM in this therapy.

### 2.3 Therapeutic regimen

The surprising result of the combined AST provided a crucial breakthrough, opening a new path in the treatment that had reached a dead end. According to the ATM drug instructions and expert consensus recommendations, ATM can be prescribed at a maximum dose of 2 g q6h to adults with life-threatening or severe *P. aeruginosa* infections. For patients with an endogenous creatinine clearance rate between 10–30 mL/min×1.73m^2^, a dosage reduction should be considered after the initial administration (Giurazza et al., 2021; Society PIAoCT, 2022).

In this case, the patient exhibited mild abnormalities in liver and renal function (**Supplementary Table 2**). Given the severity of his infection and following discussion with the clinical pharmacist, we collectively recommended changing the treatment from “cefoperazone-sulbactam (3 g q8h) + colistimethate sodium (0.15 g q12h)” to “CZA (2.5 g q8h) + ATM (2 g q6h)”. It was also advised to maintain the initial dose of ATM throughout the entire treatment course without reduction, under close monitoring of the patient’s liver and renal function. This therapeutic regimen was hereinafter referred to as “CZA + ATM_hi_”.

### 2.4 Clinical prognosis

**Figure 2:**
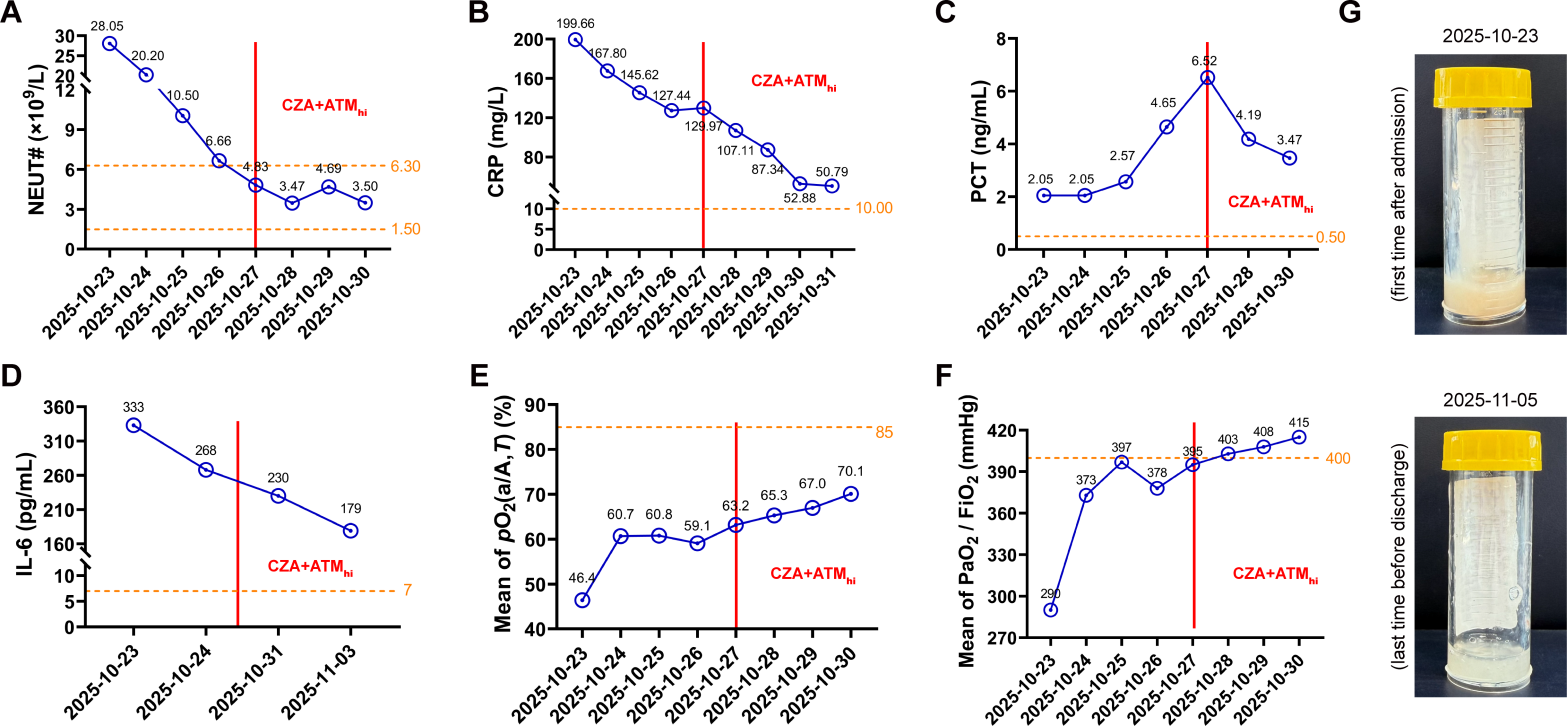
The patient received an optimistic outcome after treatments. **(A-F)** The patient’s peripheral blood neutrophil counts (NEUT#), serum concentration of C-reactive protein (CRP), procalcitonin (PCT), interleukin-6 (IL-6), mean of arterial-alveolar oxygen fraction [*p*O2(a/A,*T*)], and mean of oxygenation index (PaO_2_/FiO_2_) were measured on the indicated dates. The exact values are marked above each point. The orange dotted lines represent the edges of reference intervals. The left and right sides of the red solid line represent the periods before and after therapy of ceftazidime-avibactam (CZA) in combination with high-dose aztreonam (ATM_hi_), respectively. **(G)** The appearance of the patient’s sputum samples collected on the indicated dates.

Our suggestions were quickly adopted and eventually proved effective. After the CZA + ATM_hi_ combination, the levels of inflammatory markers in the patient’s blood decreased significantly on a daily basis (**Figures 2A–D**). His sputum changed in appearance from thick and viscous to thin and clear (**Figure 2G**). Compared to the first examination upon admission, the patient’s chest CT scans revealed a notable resolution of the inflammatory lesions across all pulmonary lobes and a significant reduction in bilateral pleural effusion (**Supplementary Video 2**). As a result, his pulmonary gas exchange function also showed obvious improvement based on the increasing means of arterial-alveolar oxygen fraction (**Figure 2E**) and oxygenation index (**Figure 2F**). After one week of therapy, the patient’s infection had been fully controlled. He was finally discharged after his lung function returned to normal.

## 3 Discussion

This case represents the first confirmed instance demonstrating that high-dose aztreonam enhanced the combined antimicrobial activity of CZA against NDM positive XDR-PA in severe pneumonia therapy. The patient in this study, presenting with advanced age, multiple underlying comorbidities, and prolonged bedrest, constituted a high-risk population for infection with hypervirulent XDR-PA strains (Li et al., 2024). After reviewing the patient’s culture records with positive results during his current hospital stay, we discovered that since the first positive culture isolated from the patient’s sputum, the XDR-PA was repeatedly detected in his peripheral blood and BALF several times, indicating the condition was growing worse. This strain is highly invasive and hard to eradicate once it infects an elderly and immunocompromised patient.

According to the latest report of antimicrobial resistance threats released by Centers for Disease Control and Prevention of the United States in July 2024, XDR-PA is regarded as a pathogen with “serious threat” level for several consecutive years, leading to over 700 million dollars attributable healthcare costs in 2017. Compared with 2019, the number of hospital-acquired XDR-PA infection cases increased by 32% in 2020, which may be associated with the prolonged length of hospital stay or secondary infections related to COVID-19 sequelae (Schwartz et al., 2024). In China, *P. aeruginosa* remains the second leading cause of hospital-acquired pneumonia (Society PIAoCT, 2022). Notably, among patients with ventilator-associated pneumonia, XDR-PA is noteworthy for its high prevalence, with isolation rates reaching nearly 30% (Yin et al., 2021). More seriously, the latest data released by China Antimicrobial Surveillance Network indicate that some XDR-PA strains have developed resistance even to novel high-level antibiotics, such as ceftolozane-tazobactam (MIC range = 0.25->128 μg/mL, MIC_50_ = 1 μg/mL, MIC_90_ = 128 μg/mL, strain number = 246, resistant rate = 13.8%) and meropenem-vaborbactam (MIC range = 0.03->64 μg/mL, MIC_50_ = 8 μg/mL, MIC_90_ = 32 μg/mL, strain number = 302, resistant rate = 28.8%) (Guo et al., 2025). This highlights the urgent need to study the responsible mechanisms and develop effective response strategies.

Findings from several recent multicenter epidemiological studies in China demonstrate that MBL production is present in over 50% of XDR-PA strains, largely comprising IMP and VIM types, with a consistently upward trend in prevalence (Wang and Wang, 2020; Xu et al., 2020). Furthermore, as a growing number of newfound MBL subtypes are identified worldwide, the situation regarding the prevention and control of XDR-PA becomes increasingly challenging (Zhai et al., 2025).

In our work, accurate carbapenemase typing was established through concordant results from the inhibitor enhancement method and lateral flow immunochromatographic assay, enabling us to select an appropriate combined AST schedule at a very early stage. The detection of NDM, a less common but significant MBL type among XDR-PA strains in China, signals its ongoing spread and emphasizes the necessity of continuous monitoring to improve patient management and infection control.

At present, cefiderocol and aztreonam-avibactam (AZA) are generally recommended as the first-line and second-line agents, respectively, against MBL producing XDR-PA on the international stage (Hidalgo-Tenorio et al., 2024). However, these two agents have only recently been approved for marketing in China. The application is limited due to the high cost and the lack of large-scale domestic data supporting their clinical efficacy. Therefore, the CZA + ATM combination regimen is currently widely adopted as an alternative in clinical practice in China. Meanwhile, clinical guidelines also specify that avibactam does not significantly enhance the bactericidal activity of ATM against CRPA (Hidalgo-Tenorio et al., 2024) — a conclusion consistent with our observations from the broth disk elution test in **Figure 1B**. Besides, a recent study demonstrated that, compared with monotherapy, the combination of CZA and ATM therapy exhibited a stronger antimicrobial effect on MBL producing CRPA *in vitro*, yielding favorable outcomes even when the strains simultaneously harbor AmpC over-expression or OprD deficiency (Montero et al., 2025).

Niklas and his colleagues recently reported that the inhibitory effect on XDR growth reaches its maximum when the concentration ratio of avibactam (AVI) to ATM is optimized at 1:4 *in vitro*. Further increasing the concentration of ATM appears to offer no additional benefit due to a saturation effect (Klein et al., 2025). In our study, we evaluated the synergistic effect of CZA (15/10 μg/mL) and ATM (30 μg/mL) using the broth disk elution method. The concentration ratio of AVI to ATM was set at 1:3 (**Figure 1B**), which was near the saturation threshold and was subsequently demonstrated to achieve favorable anti-infective outcomes in the patient.

According to the pharmacokinetic/pharmacodynamic principles, both CZA and ATM are time-dependent antibacterial agents with a short post-antibiotic effect. Therefore, to improve the probability of target attainment of the combined regimen, we extended the infusion duration of ATM from 1 h to 3 h and shortened the dosing interval from q8h to q6h. CZA and ATM were delivered via a Y-shaped infusion tube simultaneously during intravenous administration. Additionally, given the patient’s hypoalbuminemia (**Supplementary Table 2**), the concentration of free drug in the bloodstream may have been further elevated. Regarding safety, there is no incompatibility between CZA and ATM. Both agents are primarily eliminated via the kidneys as unchanged parent compounds. Thus, renal function and plasma drug concentrations were closely monitored during treatment.

Once the infection was effectively controlled, sputum or BLAF from the patient should be collected again for etiological testing, and a stepwise de-escalation therapy should be considered based on the AST results. For example, reducing the dosage of ATM for the sake of renal function protection and administering aminoglycosides or colistin via aerosol inhalation in addition to ongoing intravenous agents. Meanwhile, attention should be paid to preventing infections caused by carbapenem-resistant *Acinetobacter baumannii* complex due to prolonged ICU stay. Empirical strategies for such coverage could include, for example, high-dose ampicillin-sulbactam combined with tigecycline, minocycline, or colistin.

To summarize, we detailed the clinical features and diagnostic thinking of an elderly patient with severe pneumonia. More importantly, we also introduced an effective antimicrobial therapy against NDM positive XDR-PA, characterized by cost-effective and dose-flexible, offering additional options for personalized treatment of pulmonary infection in the future.

We attribute the successful treatment of this case to our adherence to “three actions of boldness with three principles of prudence” at several critical junctures that determined the clinical outcome. First was bold hypothesizing coupled with careful verification. The enlarged diameter of the inhibition zone between adjacent ATM disks was observed incidentally in the combined AST, which inspired us that ATM dosage might influence the synergistic effect. The conjecture was ultimately validated by the broth disk elution assay (**Figure 1**). Second was bold recommendations paired with rigorous oversight. We strictly followed the treatment indications by fully integrating the combined AST results, drug instructions, and expert consensus statements. After a comprehensive assessment of the patient’s condition in collaboration with clinical pharmacists (**Supplementary Table 2**), we proposed a novel therapeutic regimen, namely CZA + ATM_hi_, and recommended its clinical use for the patient’s anti-infective treatment. Third was bold implementation paired with meticulous assessment. Given the recurrent infection and critical condition of the patient, a tripartite agreement was reached among clinicians, clinical pharmacists, and microbiologists in our team. We ultimately decided to proceed with CZA + ATM_hi_ combination therapy under the close surveillance of the patient’s liver and renal function (**Supplementary Figure 3**), while timely evaluating the anti-infective effect (**Figure 2**). Through these collaborative efforts, CZA and ATM worked like two sharp swords, striking together with a remarkable synergistic antibacterial effect against the NDM positive XDR-PA. Finally, the patient exhibited a favorable clinical response and was successfully rescued from the critical state.

## Data Availability

The original contributions presented in the study are included in the article/**Supplementary Material**. Further inquiries can be directed to the corresponding author.

## Supplementary material

Supplementary Figure 1 Pathogenic evidence causing pulmonary infection in patient. **(A)** A representative image of white blood cells (hollow star) and *Pseudomonas aeruginosa* (solid arrow) in the sputum smear was captured under the optical microscope after Gram staining. The upper right corner shows the partial magnification of the section marked with a dashed box. Bar, 20 μm. **(B)** A large quantity of highly purified *Pseudomonas aeruginosa* was isolated from the patient’s sputum using different culture mediums. **(C)** The mass spectral profiles of *Pseudomonas aeruginosa* acquired by matrix-assisted laser desorption ionization-time of flight mass spectrometry. The horizontal and vertical coordinate represents mass-to-charge ratio (m/z) and relative intensity of ion current (%), respectively.

Supplementary Figure 2 Carbapenemase detection of *Pseudomonas aeruginosa*. The phenotype and genotype of carbapenemase produced by *Pseudomonas aeruginosa* were determined by **(A)** the carbapenemase inhibitor enhancement method and **(B)** the lateral flow immunochromatographic assay, respectively. Abbreviations: IPM, imipenem; APB, 3-aminophenylboronic acid; EDTA, ethylenediaminetetraacetic acid; N, NDM; I, IMP; V, VIM; O, OXA-48-like; K, KPC; C, validity control.

Supplementary Figure 3 The patient’s blood biochemical parameters before and after the combined medication of ceftazidime-avibactam and high-dose aztreonam. The patient’s **(A–C)** liver and **(D–F)** kidney function were assessed via blood tests on the indicated dates. The exact values are marked above each point. The orange dotted lines represent the edges of reference intervals. The left and right sides of the red solid line represent the periods before and after combination therapy, respectively. Abbreviations: ALB, serum albumin; ALT, alanine transaminase; AST, aspartate transaminase; BUN, blood urea nitrogen; Ccr, endogenous creatinine clearance rate; Cys C, cystatin C; CZA, ceftazidime-avibactam; ATM_hi_, high-dose aztreonam.

Supplementary Table 1 Antimicrobial susceptibility profile of *Pseudomonas aeruginosa*.

Supplementary Table 2 Assessment of the patient’s liver and kidney function two days prior to combination therapy.

Supplementary Video 1 The initial computed tomography scan of the patient’s chest after admission. The examination was performed using a 128-row multidetector in the transverse plane without contrast. The serial images were obtained with a 1.25 mm slice thickness and displayed at 15 frames per second.

Supplementary Video 2 The continuous computed tomography scan of the patient’s chest one week after the combination use of ceftazidime-avibactam and high-dose aztreonam. The examination was performed using a 128-row multidetector in the transverse plane without contrast. The serial images were obtained with a 1 mm slice thickness and displayed at 20 frames per second.
