# Supplementary material for: High-dose aztreonam potentiates the combined antimicrobial activity of ceftazidime-avibactam against extensively drug-resistant New Delhi metallo-β-lactamase positive Pseudomonas aeruginosa in severe pneumonia treatment: the first global case report
Source: Front Cell Infect Microbiol. 2026 Mar 4;16:1786251. doi: 10.3389/fcimb.2026.1786251 (PMC12996157; doi:10.3389/fcimb.2026.1786251)
Supplement: Supplementary Figure 1 — Pathogenic evidence causing pulmonary infection in patient. (A) A representative image of white blood cells (hollow star) and Pseudomonas aeruginosa (solid arrow) in the sputum smear was captured under the optical microscope after Gram staining. The upper right corner shows the partial magnification of the section marked with a dashed box. Bar, 20 μm. (B) A large quantity of highly purified Pseudomonas aeruginosa was isolated from the patient’s sputum using different culture mediums. (C) The mass spectral profiles of Pseudomonas aeruginosa acquired by matrix-assisted laser desorption ionization-time of flight mass spectrometry. The horizontal and vertical coordinate represents mass-to-charge ratio (m/z) and relative intensity of ion current (%), respectively. [file DataSheet1.docx]

Supplementary Material

High-dose aztreonam potentiates the combined antimicrobial activity of ceftazidime-avibactam against extensively drug-resistant New Delhi metallo-β-lactamase positive *Pseudomonas aeruginosa* in severe pneumonia treatment: the first global case report

Yifeng Liu^1,2^, Cuiju Mo^1,2^, Meng Li^1,2*^

^1^ Department of Clinical Laboratory, The First Affiliated Hospital of Guangxi Medical University, Nanning, China

^2^ Key Laboratory of Clinical Laboratory Medicine of Guangxi Medical University, Education Department of Guangxi Zhuang Autonomous Region, Nanning, China

*** Correspondence:**Meng Li
gxmulimeng@foxmail.com.

**Supplementary Table 1. Antimicrobial susceptibility profile** **of *Pseudomonas aeruginosa*.**

| **Antibacterial agent** | **Inhibition zone diameter (mm)** | **Minimum inhibitory concentration (μg/mL)** | **Interpretation** |
| --- | --- | --- | --- |
| Piperacillin-tazobactam | 10 |  | Resistant |
| Ticarcillin-clavulanate |  | >=128 | Resistant |
| Ceftazidime |  | >=64 | Resistant |
| Cefepime |  | >=32 | Resistant |
| Aztreonam | 6 |  | Resistant |
| Imipenem | 6 |  | Resistant |
| Meropenem |  | >=16 | Resistant |
| Ciprofloxacin |  | >=4 | Resistant |
| Levofloxacin |  | >=8 | Resistant |
| Tobramycin |  | >=16 | Resistant |
| Colistin |  | 2 | Intermediate |

Footnote: The inhibition zone diameters and minimum inhibitory concentrations were determined by the Kirby-Bauer disk diffusion test and broth microdilution method, respectively. The interpretation was made according to the breakpoints in the M100 guideline (35^th^ Edition, Table 2B-1) issued by Clinical and Laboratory Standards Institute.

**Supplementary Table 2. Assessment of the patient’s liver and kidney function two days prior to combination therapy.**

| **Index** | **Value**  **(Oct. 25)** | **Trend**  **(Oct. 25)** | **Value**  **(Oct. 26)** | **Trend**  **(Oct. 26)** | **Unit** | **Reference interval** |
| --- | --- | --- | --- | --- | --- | --- |
| ALB | 29.1 | L | 31.0 | L | g/L | 40.0-55.0 |
| AST | 164 | H | 168 | H | U/L | 15-40 |
| ALT | 30 | N | 30 | N | U/L | 9-50 |
| BUN | 28.01 | H | 22.85 | H | mmol/L | 3.60-9.50 |
| Scr | 194 | H | 200 | H | μmol/L | 57-111 |
| Ccr | 25.14 | L | 24.39 | L | ml/min×1.73m^2^ | 85.00-125.00 |
| Cys C | 4.29 | H | 3.26 | H | mg/L | 0.59-1.03 |

Abbreviations: ALB, serum albumin; ALT, alanine transaminase; AST, aspartate transaminase; BUN, blood urea nitrogen; Scr, serum creatinine; Ccr, endogenous creatinine clearance rate; Cys C, cystatin C; H, above the upper limit of the reference interval; L, below the lower limit of the reference interval; N, within the reference interval.


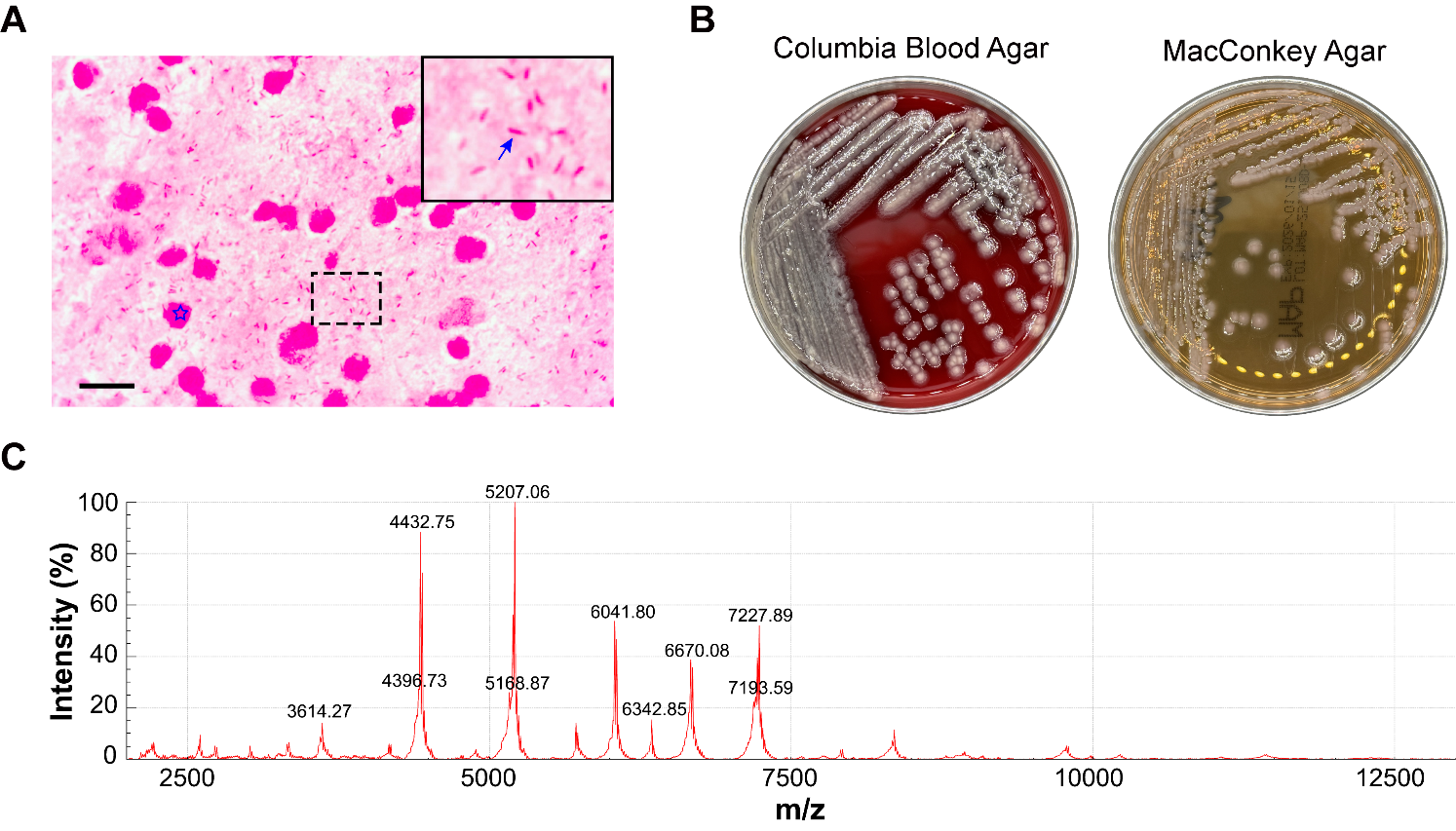


**Supplementary Figure 1. Pathogenic evidence causing pulmonary infection in patient.**

**(A)** A representative image of white blood cells (hollow star) and *Pseudomonas aeruginosa* (solid arrow) in the sputum smear was captured under the optical microscope after Gram staining. The upper right corner shows the partial magnification of the section marked with a dashed box. Bar, 20 μm. **(B)** A large quantity of highly purified *Pseudomonas aeruginosa* was isolated from the patient’s sputum using different culture mediums. **(C)** The mass spectral profiles of *Pseudomonas aeruginosa* acquired by matrix-assisted laser desorption ionization-time of flight mass spectrometry. The horizontal and vertical coordinate represents mass-to-charge ratio (m/z) and relative intensity of ion current (%), respectively.


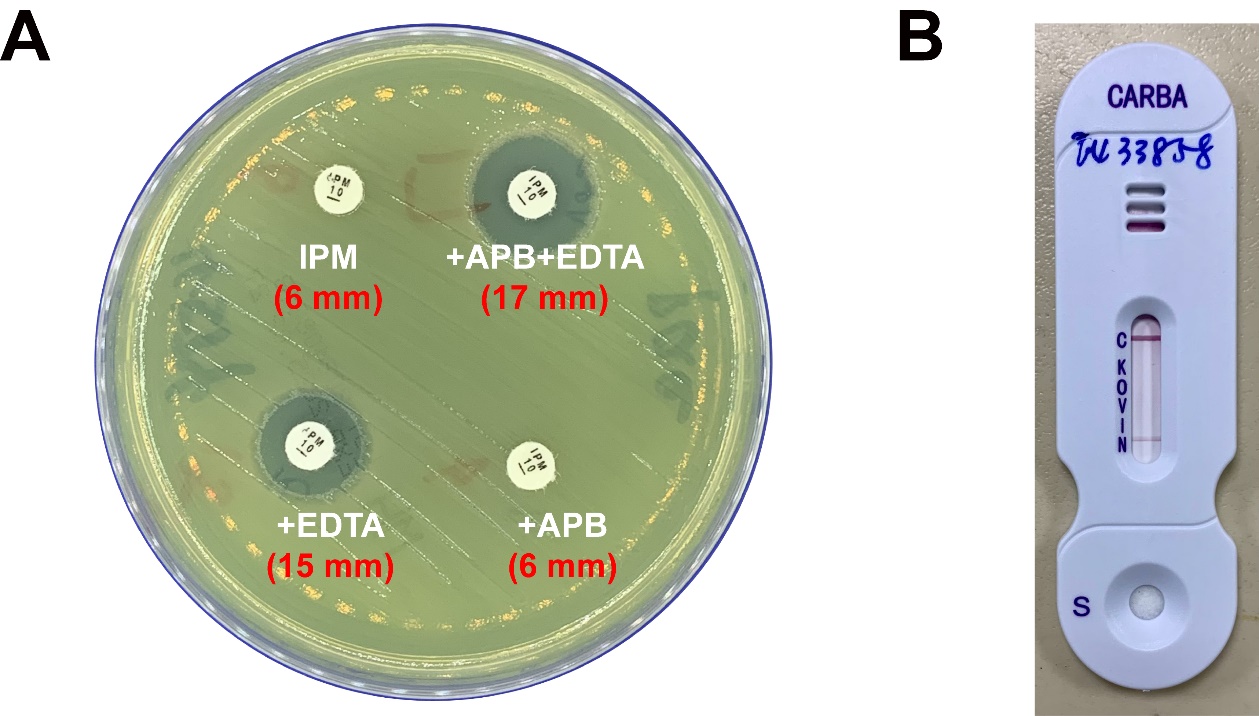


**Supplementary Figure 2. Carbapenemase detection of *Pseudomonas aeruginosa*.**

The phenotype and genotype of carbapenemase produced by *Pseudomonas aeruginosa* were determined by **(A)** the carbapenemase inhibitor enhancement method and **(B)** the lateral flow immunochromatographic assay, respectively. Abbreviations: IPM, imipenem; APB, 3-aminophenylboronic acid; EDTA, ethylenediaminetetraacetic acid; N, NDM; I, IMP; V, VIM; O, OXA-48-like; K, KPC; C, validity control.

**
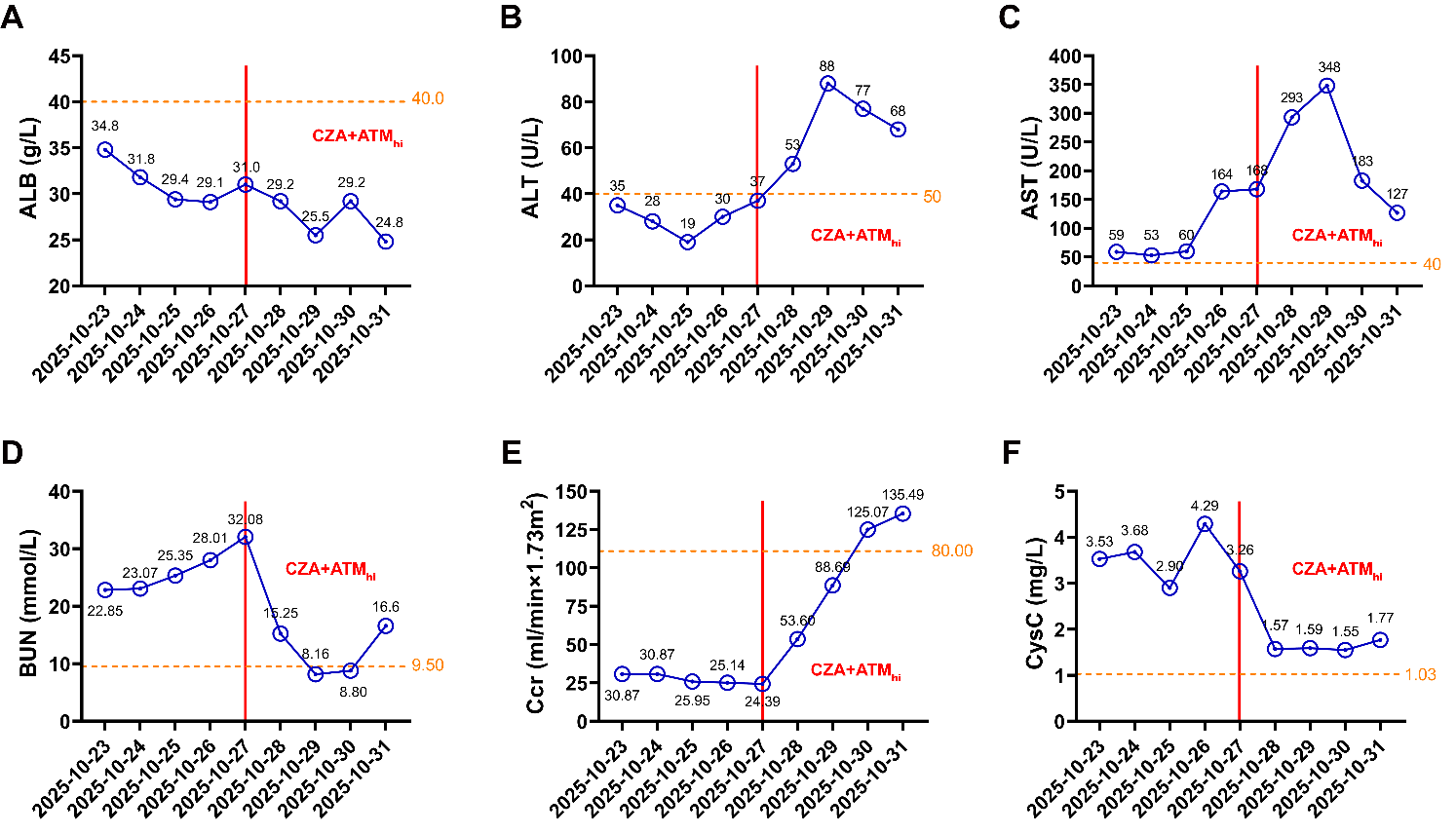
**

**Supplementary Figure 3. The patient’s blood biochemical parameters before and after the combined medication of ceftazidime-avibactam and high-dose aztreonam.**

The patient’s (A-C) liver and (D-F) kidney function were assessed via blood tests on the indicated dates. The exact values are marked above each point. The orange dotted lines represent the edges of reference intervals. The left and right sides of the red solid line represent the periods before and after combination therapy, respectively. Abbreviations: ALB, serum albumin; ALT, alanine transaminase; AST, aspartate transaminase; BUN, blood urea nitrogen; Ccr, endogenous creatinine clearance rate; Cys C, cystatin C; CZA, ceftazidime-avibactam; ATM_hi_, high-dose aztreonam.

**Supplementary Video 1. The initial computed tomography scan of the patient’s chest after admission.**

The examination was performed using a 128-row multidetector in the transverse plane without contrast. The serial images were obtained with a 1.25 mm slice thickness and displayed at 15 frames per second.

**Supplementary Video 2. The continuous computed tomography scan of the patient’s chest one week after the combination use of ceftazidime-avibactam and high-dose aztreonam.**

The examination was performed using a 128-row multidetector in the transverse plane without contrast. The serial images were obtained with a 1 mm slice thickness and displayed at 20 frames per second.
